# Computational Predictions for Single Chain Chalcogenide-Based One-Dimensional Materials

**DOI:** 10.3390/nano7050115

**Published:** 2017-05-17

**Authors:** Blair Tuttle, Saeed Alhassan, Sokrates Pantelides

**Affiliations:** 1 Department of Physics and Astronomy, Vanderbilt University, Nashville, TN 37235, USA; pantelides@vanderbilt.edu; 2 Department of Physics, Penn State Behrend, Erie, PA 16563, USA; 3 Department of Chemical Engineering, The Petroleum Institute, P.O. Box 2533, Abu Dhabi, UAE; salhassan@pi.ac.ae; 4 Department of Electrical Engineering and Computer Science, Vanderbilt University, Nashville, TN 37235, USA

**Keywords:** nanowires, chalcogenides, ab initio calculations

## Abstract

Exfoliation of multilayered materials has led to an abundance of new two-dimensional (2D) materials and to their fabrication by other means. These materials have shown exceptional promise for many applications. In a similar fashion, we can envision starting with crystalline polymeric (multichain) materials and exfoliate single-chain, one-dimensional (1D) materials that may also prove useful. We use electronic structure methods to elucidate the properties of such 1D materials: individual chains of chalcogens, of silicon dichalcogenides and of sulfur nitrides. The results indicate reasonable exfoliation energies in the case of polymeric three-dimensional (3D) materials. Quantum confinement effects lead to large band gaps and large exciton binding energies. The effects of strain are quantified and heterojunction band offsets are determined. Possible applications would entail 1D materials on 3D or 2D substrates.

## 1. Introduction

Thin films, including few atomic layers and monolayers, grown on substrates have been studied for decades using methods such as molecular beam epitaxy, pulsed laser deposition, or even simple oxidation. A breakthrough idea by Novoselov and Geim [1] in 2004 to exfoliate and study a monolayer from a layered material, namely graphite, led to the explosive growth of new research into two-dimensional (2D) materials, which now includes free-standing monolayers, bilayers, etc., fabricated in a variety of ways, but also monolayers and bilayers on substrates. The number of 2D materials with unique and unusual properties has grown rapidly and has generated enormous interest from both a basic [2,3,4,5] and applied [3,6,7] research perspective. Two-dimensional materials are promising for electronic [8], optical [9], catalytic [10] and other applications. Given the maturity of techniques to grow and examine 2D materials and devices [11,12,13,14], we turn our attention to analogous one-dimensional (1D) systems.

Since at least the discovery of carbon nanotubes, there has been great interest in material systems in which electrons mainly propagate in only one dimension. For carbon nanotubes, semiconducting or metallic tubes are possible depending on a tube’s bonding topology. Boron-nitride nanotubes [15] and other nanotubes have been fabricated and studied. Moreover, wires with nanometer scale diameters have also been grown using Si [16], Se [17] and GaAsSb [18], to name a few. One-dimensional materials have been used in a variety of two- and three-terminal electrical devices [11,13]. Recently, sub-nanometer scale wires were sculpted from 2D monolayer transition-metal dichalcogenides [8]. As scaling proceeds, materials and devices made from single chain 1D systems are becoming realizable.

In chemistry of course, there exist many molecular monomers, which can be combined to form single chain oligomers or polymers. Indeed, there has been a long history of theoretical studies of molecular chains, including both finite and infinite systems [19,20,21,22]. There is continued interest in polymer chains and crystalline polymers. For instance, recent studies of crystalline polymers examined dielectric properties for a large number of carbon-based polymers [23]. Many infinite chains are known to be stable. Early studies of S and Se chains explored the electronic and structural properties and provided a fundamental understanding of these 1D systems [21]. Also, early theoretical studies of polyacene and sulfur nitride infinite chains elucidated the nature of their superconducting properties [19,24]. Motivated by the emerging possibility of 1D materials for a wide variety of applications, we present new state-of-the-art theoretical results for chalcogenide-based 1D materials.

In this paper, we apply modern electronic structure methods to calculate a range of properties of selected 1D materials. Given the recent interest in chalcogenide-based 2D materials [2,25,26], here we consider chalcogenide-based 1D systems. Specifically, we examine individual chains of chalcogens (S, Se, Te) and of silicon dichalcogenides (SiS_2_ and SiSe_2_), all of which are insulating. In addition, we consider a single strand of SNH, which is insulating, and a strand of S–N, which is metallic. Finally, for comparison, we include infinite carbon-based chains of polyphenylene (C_6_H_4_) [27] and polyacene (C_4_H_2_) [19,20], which can be viewed as a single strand cut from monolayer graphene. Carbon systems are included since these systems are well understood experimentally and theoretically and so provide a good benchmark. We find that chalcogenide-based1D materials can in principle be exfoliated from three-dimensional (3D) polymeric crystals with relative ease (i.e., exfoliation energies are relatively small). While growth mechanisms are not explicitly calculated, as new 1D materials are grown, experimentalists can use the present results to understand the fundamental versus the growth-dependent electrical/mechanical properties. Quantum confinement effects lead to increased band gaps and larger exciton binding energies when compared to their bulk counterparts. We also calculate strain moduli and the deformation potentials for electrons and holes of these 1D materials. Electronic band offsets are reported for select heterojunctions. The properties considered are critical for applications and the prospects for applications are discussed in the context of the new results. Finally, these results should help in the selection of materials to be synthesized based on 1D applications of interest.

## 2. Results

To examine the properties of 1D materials, we begin with unit cells of the corresponding bulk orthorhombic crystals. The equilibrium lattice constant of an isolated 1D chain is found by allowing the chain length and atomic positions to relax until a minimum energy structure is found. In Table 1, the 1D lattice constants (*a_o_*) are reported. The values are close to previously reported values and/or to the c-axis of the corresponding orthorhombic 3D crystal.

**Figure 1:**
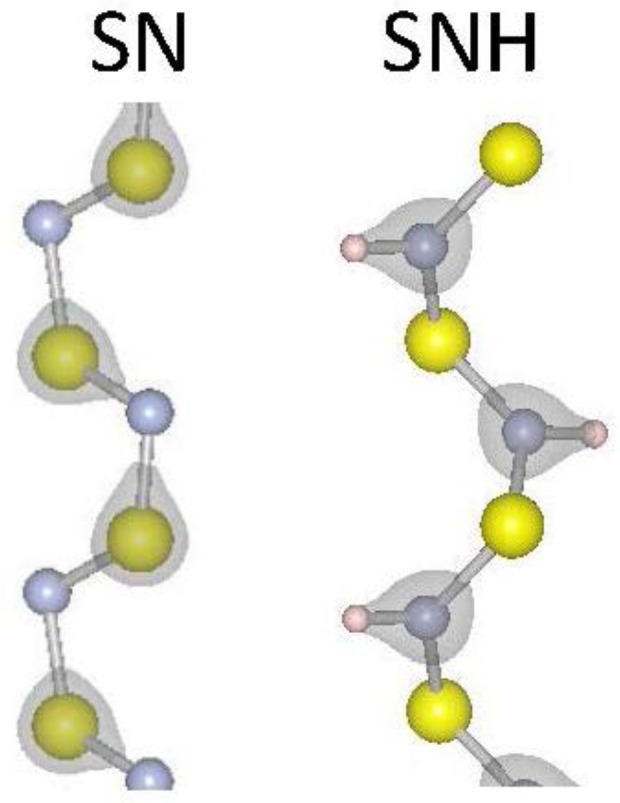
Atomic and electronic structure for S–N and SNH_2_ one-dimensional (1D) systems. Grey regions represent surfaces of constant charge density. The charge density isosurface is for 2.3 × 10^−4^ electrons per Å^3^. Large yellow, small grey and small white spheres represent S, N and H, respectively.

The mechanical strain modulus in one dimension is defined as

(1) $$E_{sm}=\frac{1}{L}\frac{d^{2}U}{d\epsilon^{2}}$$

where *L* is the periodic length of the one-dimensional material, *U* is the total energy of the material, and dϵ=dx/L is the differential strain factor. The strain modulus (*E_sm_*) is a measure of the energy needed to vary the lattice constant of the material. We have calculated the strain moduli for our selected 1D materials and report the results in Table 1. The strain modulus results show a clear trend for materials that contain chalcogens: going down the periodic column, from S to Se to Te, the strain modulus becomes smaller, indicating weaker covalent bonding as expected. A similar but less pronounced effect is found comparing SiS_2_ to SiSe_2_ results. As expected, the sp^3^ orbitals of Si enhance covalent bonding with chalcogenides. The carbon chains considered (C_6_H_4_, C_6_H_4_) have notably higher strain moduli due to their strongly covalent C–C bonds. It is notable that hydrogenation of S–N results in a lower strain modulus, suggesting that the inclusion of hydrogen weakens the S–N bonding. Figure 1 reports the iso-surfaces of the electronic charge density for S–N and SNH. Indicative of a weakening S–N bond, the charge moves from around the S atoms in S–N to between the N and H atoms in SNH. To be more quantitative, the charge at the mid-point of a S–N bond is 16% larger in 1D S–N than in 1D SNH. Finally, tellurium is the most ductile material considered here and would be attractive for device applications where strain is inherently a part of fabrication and operation.

In principle, some of the 1D materials considered here can be isolated using the corresponding 3D materials and exfoliation techniques. The exfoliation energy is an important quantity for understanding the physics of a layered material [28]. The exfoliation energies has been calculated by comparing the energy per atom for the single chain to the bulk material. While van der Waals interactions are difficult to capture theoretically, the results reported in Table 1 help us understand the trends and electronic structure of these materials (here we consider a hypothetical bulk form of S similar to that of Se and Te for comparison purposes since the room-temperature form of 3D S comprises sulfur rings). The pure chalcogenide systems display the expected exfoliation trends, with S having the lowest and Te the highest exfoliation energy. This trend is expected since covalency decreases from S to Se to Te, and therefore electrons are free to participate in inter-chain bonding. To explore the nature of the inter-chain bonding in the pure chalcogenides, we fix the 1D and 3D coordinates found with van de Waals interactions and then turn off the van de Waals interaction. The exfoliation energies dramatically decrease, suggesting the inter-chain interaction is mainly van de Waals in character. The silicon dichalcogenides exhibit a similar but moderate trend, with values similar to S. Hydrogenated S–N has a larger exfoliation energy than pure S–N, which has the lowest exfoliation energy of the systems considered here. Apparently, the dipole of the N–H bond produces a larger van der Waals effect than the metallic electrons of the pure S–N. The carbon chains considered (C_6_H_4_, C_6_H_4_) have exfoliation in the middle of the range found in the present materials. The exfoliation energies reported in Table 1 are only slightly larger than the value for graphene.

**Figure 2:**
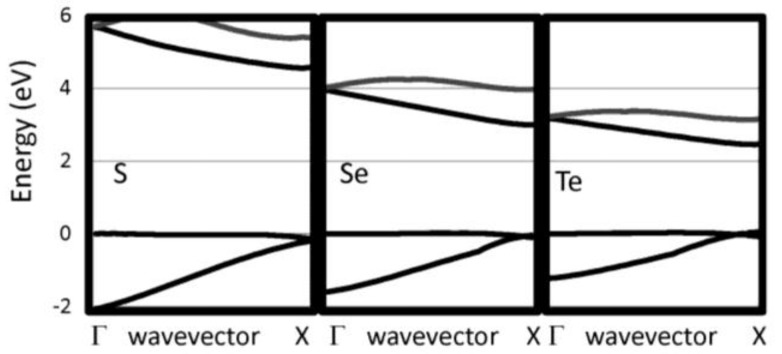
Near gap bands are reported for several 1D insulating systems, including S, Se, and Te.

**Figure 3:**
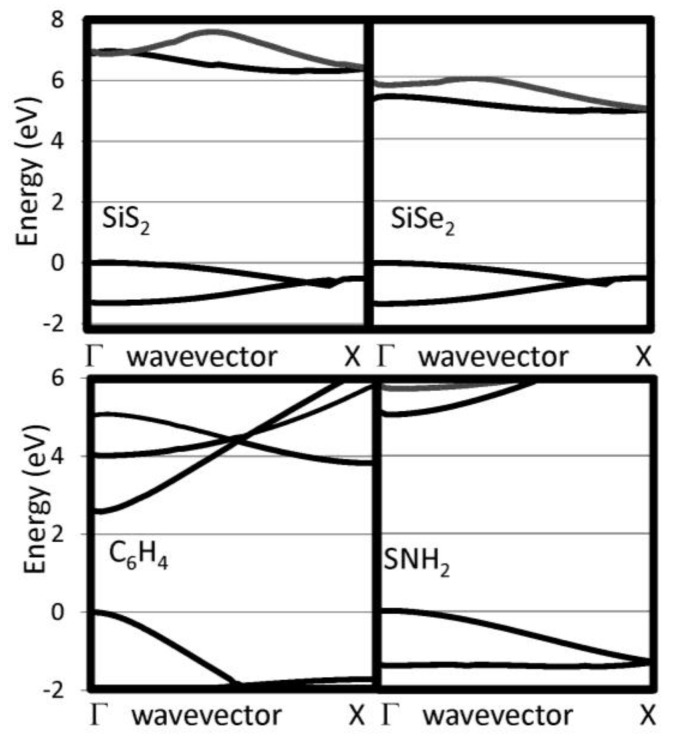
Near gap bands are reported for several 1D insulating systems, including SiS_2_, SiSe_2_, C_6_H_4_ and SNH_2_.

The G_0_W_0_ band structures have been calculated for our selected 1D chains. We have checked that the bands perpendicular to the chain direction are flat indicating that the supercell used are sufficiently large to eliminate interactions between neighboring chain. The 1D band structures for pure chalcogenides and the other insulating systems are reported in Figure 2 and Figure 3, respectively. The band gaps are reported in Table 1. For the pure chalcogens, the valence band gap is very flat. Sulfur and selenium 1D chains both have valence band maxima at G which is only 0.13 eV and 0.05 eV, respectively, higher than at X. For all pure chalcogenides, the conduction band minimum is at the X point. For the silicon dichalcogenides, the minimum gap is indirect with the valence band maximum at Γ and the conduction band minimum near X. In Table 1, both the direct and indirect minimum gaps are reported in cases where the minimum gap is indirect. The hydrogenated chains, polyphenylene and sulfur nitride, have direct minimum gaps at Γ. The pure chalcogenides are semiconducting with wide band gaps of 4.73, 3.05 and 2.41 eV for S, Se and Te, respectively. The band gap for the chalcogenides are lower as the materials covalency increases. Polyphenylene has a gap slightly larger than sulfur. The dichalcogenides (SiS_2_ and SiSe_2_) and SNH are significantly more insulating materials. Generally, 1D chains have larger band gaps and lower dielectric constants than their bulk counterparts. The effect is less dramatic, however, for the more insulating materials.

**Figure 4:**
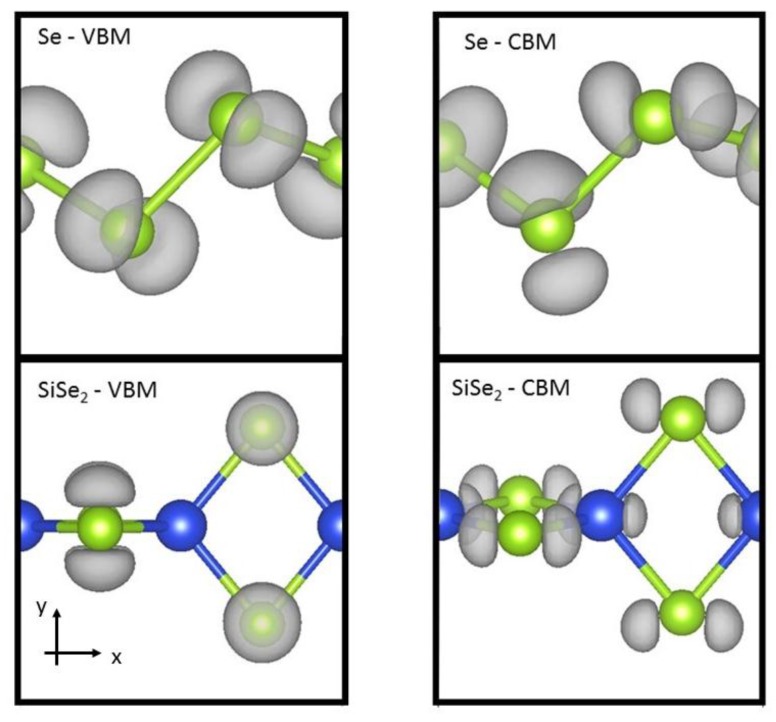
Atomic and electronic structures are reported for Se (SiSe_2_) valence band maximum (VBM) and conduction band minimum (CBM). Grey regions represent surfaces of constant charge density. Large dark (blue) and small light (green) spheres represent Si and Se, respectively.

To understand the electronic structure of the materials, we examine the charge densities in a couple cases. In Figure 4, the charge density isosurfaces are reported for the valence band maximum and conduction band minimum states of Se and SiSe_2_. The charge isosurfaces are similar for Se and SiSe_2_, and these plots are representative of the isosurfaces of the other 1D systems studied here. The charge density isosurface for the valence band maximum state indicates that charge distributes perpendicular to the plane of the Se zig-zag bonding. The valence band maximum state is characterized by a non-bonding p-orbital localized on the chalcogen atom. This behavior is natural for chalcogen atoms with two strong covalent bonds. Figure 4 also shows that the conduction band minimum state has charge localization on the chalcogen atom with p-orbital along *x*-direction where the bond is along the *x*–*y* direction (see Figure 4). In the silicon dicalchogenides, there is also some p-orbital charge localization on the silicon atom. The charge localization reported here are consistent with previous analysis for S and Se chains [21].

**Figure 5:**
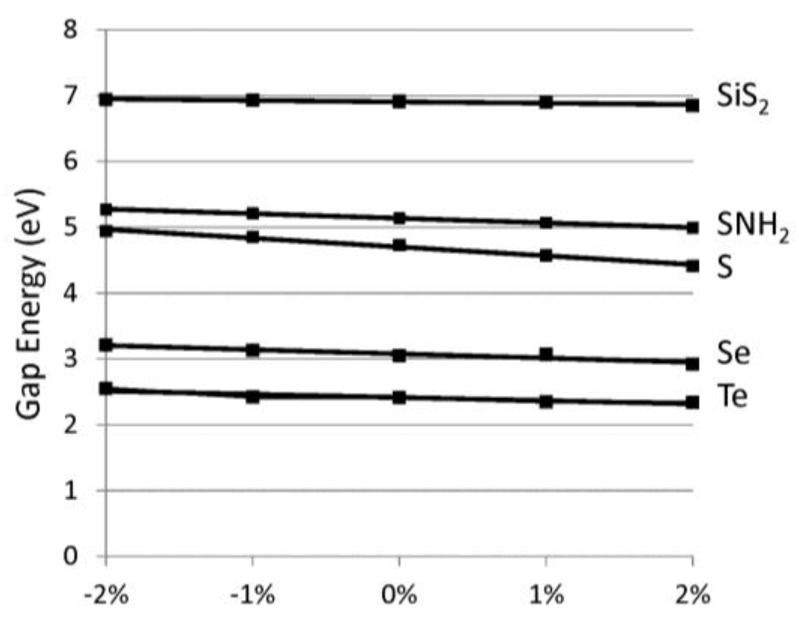
Direct band gap energy (eV) versus relative % strain, as defined in text. The chalcogen-based systems have positive slopes and are represented by solid lines whereas C_6_H_4_ has a negative slope and is represented by a dashed line.

Applying these chains in opto-electronic devices would require a consideration of the effects of strain on the electronic properties. The band gap versus lattice strain is reported in Figure 5 for selected chalcogenide-based 1D materials. Here, we focus on direct gap variations, which are more important for optical applications. We find the variation of gap to strain is linear. Table 1 reports the slope of the gap variation with strain. The relative % strain is defined as 100 × *Δa/a_o_* where *Δa* difference between the strained lattice value and *a_o_*, the minimum lattice. The band gaps vary by about 0.1 eV for each 1% of lattice strain. For the chalcogens, compressive strain (although difficult to realize) would increase the band gaps whereas tensile strain would lower the band gaps. We find most of the band gap change is due to the variation in the conduction band minimum. This is reasonable since the valence band edge wavefunctions are non-bonding p-orbitals (see Figure 4), which are insensitive to variations in the bond lengths. The polyphenylene (C_6_H_4_) chains (dashed line) displays the opposite band gap trend with strain because of its different chemical bonding. Specifically, we find that, for polyphenylene, most of the lattice strain is accommodated by the single linkage C–C bond whereas in the other cases strain is accommodated symmetrically throughout the system. The variation in band edges with strain would be important when considering heterojunction device applications where significant strain can be expected. Combining the strain moduli results above and these deformation results, one can determine the strain necessary to modify the band edges by a desired amount.

**Figure 6:**
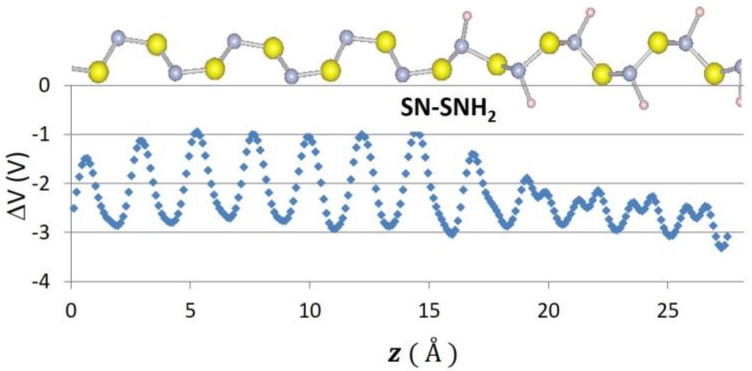
Atomic positions and change in local potential (eV) as a function of z-distance, in Å for Se–Te.

**Figure 7:**
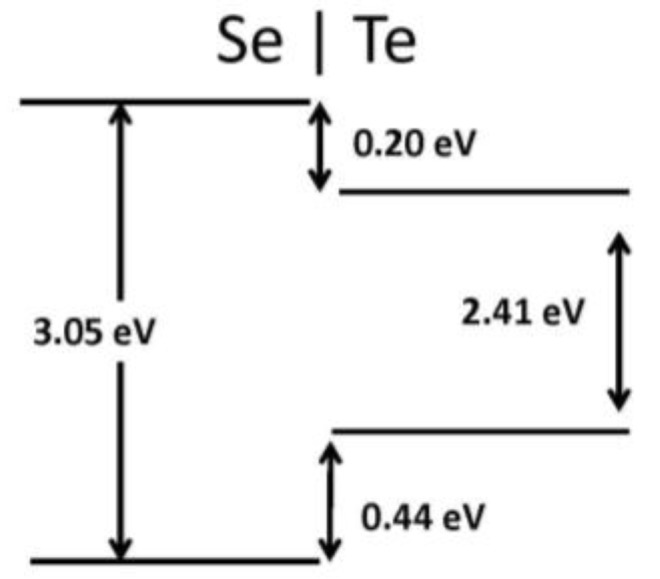
Heterojunction band diagram for the Se–Te system.

**Figure 8:**
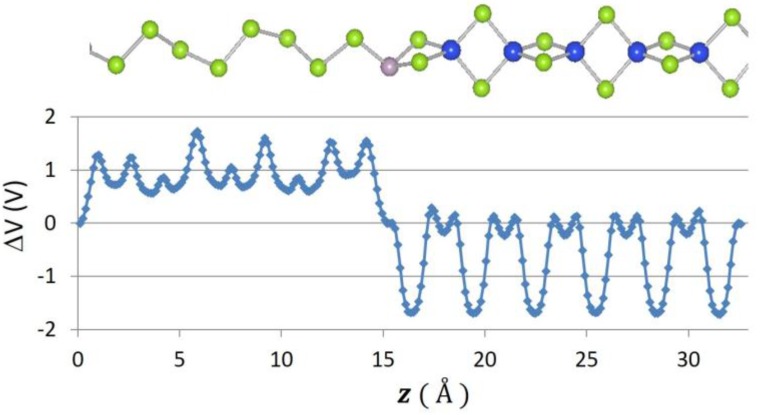
Atomic positions and change in local potential (eV) as a function of *z*-distance, in Å for the Se–P–SiSe_2_ junction.

Recently, 2D graphene nanoribbon heterojunctions have been grown and examined experimentally [16] while 2D metal dichalcogenide heterojunctions have been examined theoretically [25]. In Table 2, we report predictions for the band offset of Te–Se, SiSe_2_–SiS_2_, and SN–SNH 1D heterojunctions. The structure and the planar averaged local potential (*ΔV*) are reported in Figure 6 for the Se–Te heterojunction while in Figure 7 we report the interfacial band diagram for the Se–Te heterojunctions. The Se–Te and SiS_2_–SiSe_2_ heterojunctions are non-polar. The SN–SNH junction has a polar interface. The local potential therefore has a linear slope away from the interface dipole. Subtracting interfacial dipole effect, we determine the SN–SNH band offset reported in Table 2. The conduction band offset for SN–SNH is 3.21 eV, which is also the Schottky barrier for this metal–insulator system. While lattice mismatch is a concern for even 2D heterojunctions [2], single-chain 1D materials do not suffer this limitation although junction atoms maybe needed in order to connect two chains which have differing bonding topologies. For instance, phosphorous can be used to connect 1D Se and SiSe_2_ chains as illustrated in Figure 8. One can use these heterojunction band offset results to assess the potential of extended 1D junctions for a variety of applications. We find the Se–Te would not be suitable for semiconductor-insulator applications because the offsets are too small but the interface of Se–P–SiSe_2_ may be useful for electron quantum wells. Overall, given the lack of lattice mismatch and their range of band gaps, 1D chalcogenide-based heterojunctions are promising for a variety of applications.

## 3. Discussion

Exfoliation techniques may be used to isolate these chalcogenide-based 1D materials. A procedure may start from the adhesive exfoliation originally used to isolate graphene [29]. This method has been optimized and applied to other 2D systems [30]. Once 2D materials have been isolated, one may be able to use a transmission electron microscope (TEM) to isolate an arbitrary-length single strand, creating a 1D system. This TEM technique has long has been used to create single-strand chains of carbon atoms from graphene [31]. Another possibility may be to grow single chains on surfaces using molecular beam epitaxy, or other growth techniques, as has been implemented to construct 1D structures of GaAs [32].

Quantum confinement effects are important for predicting the electronic structure of 1D materials. Selenium is a case in point. In bulk crystalline Se, we calculate the minimum direct band gap to be 1.62 eV, close to the experimental estimate of 1.8 eV [33]. The diagonal components of the dielectric tensor are ε_perp_ ~12 and ε_para_ ~ 20. These large dielectric constants result in a small exciton binding energy ~ 0.02 eV for the bulk material. In contrast, a 1D chain of Se has a band gap of 3.05 eV, almost double the bulk value. Quantum confined electrons result in large excitonic in the 1D systems. For instance, 1D Se has a large exciton binding energy, ~1.7 eV. For 1D Se, the G_0_W_0_ direct gap is 3.05 eV but when excitonic effects are included the direct gap reduces to 1.34 eV. Therefore, 1D Se would make a good absorber in the visible range. By a similar analysis, we find that pure 1D Te would be useful as an infra-red (IR) absorber with an optical gap of 0.9 eV. Excitonic effects are smaller for the more insulating 1D chains.

When considering 1D single-chain materials for applications, we should take a new look at old materials. For instance, selenium has had a long history in semiconductor device applications. Selenium rectifiers are ubiquitous in antique radios. More recently, there has been interest in the electrical and optical properties of amorphous selenium (a-Se) thin films for use in photoconductor devices [34,35]. One-dimensional selenium chains combined with plasmonic materials could also serve as efficient photoconductors in nano-opto-electric devices similar to the current use of 2D MoS_2_ with plasmonics [9].

Lasers may also be a promising application of 1D chalcogenide-based materials. The large exciton binding energies (mentioned above) make these materials good candidates for optically pumped lasers. Decades ago, one-dimensional GaAs quantum wires were used for laser applications [36]. Over the years, advances in growth techniques have reduced the diameter of such quantum wire systems [32,37]. The use of carbon nanotubes for lasing systems has also been achieved [38]. Based on the band-offsets reported here, chalcogenide-based 1D heterojunction systems are promising for optically pumped single-chain lasers.

## 4. Methods

We employ density functional theory (DFT) [39,40] and examine the properties of these materials using the VASP computer code [41,42]. A plane-wave basis with a cutoff energy of ~300 eV was used based on the standard projected augmented wave (PAW) potentials to treat core electrons [43,44]. Calculations were performed with the generalized-gradient corrected functional of Perdew, Becke and Ernzerhof (PBE) [45] to treat exchange and correlation. In addition, for bulk calculations, van der Waals (vdW) interactions were treated in a semi-empirical fashion using the method of Grimme [46]. All atomic coordinates and lattice parameters were relaxed to their equilibrium values at T = 0 K using a force tolerance of 0.01 eV/Å.

Single strands are isolated using supercells including a vacuum of ~1.0 nm. Tests with a larger vacuum at ~2 nm found no significant changes to the quantities reported here. We have calculated the mechanical properties at the PBE level of theory. For electronic structure, we use the non-self-consistent G_0_W_0_ approximation [47,48] which accounts for the many-body electron interactions but retains the input PBE wavefunctions. The G_0_W_0_ quasi-particle electronic bands are calculated for a 75 × 1 × 1 k-point mesh. From the G_0_W_0_ quasi-particle bands, we calculate excitonic effects with the Bethe–Salpeter equation (BSE) which accounts for the interaction of a quasi-electron and a quasi-hole. The present calculations employ the Tamm–Dancoff approximation as implemented in the Vienna Ab initio Simulation Package (VASP) [26]. The four highest valence bands and the four lowest conduction bands were used as a basis for excitonic eigenstates. Employing the BSE, we calculate direct excitation energies and intensities, which can be compared with optical absorption experimental data. Heterojunction offsets are calculated in the standard band alignment method [49,50] using PBE for the interface potential alignment and, consistent with recent advances [51], we employ the G_0_W_0_ results for the bulk band edges.

## 5. Conclusions

Innovation in the growth and design of 2D materials motivated the present exploration of extended 1D materials. Specifically, there is interest in chalcogenide-based thin films including 2D materials (e.g., SiSe). Techniques for creating molecular-scale transistors from single nanotubes [38] and other similar advances also motivates the search for single chain 1D materials that may also be useful in such applications. 

In this study, we calculate the properties of chalcogenide-based 1D materials including S, Se and Te along with silicon-dichalcogenides (SiS_2_ and SiSe_2_) and sulfur nitrides (S–N and SNH). The main results of our study are presented in Table 1 where we report the strain moduli, the exfoliation energies, band gaps, and gap deformation potentials. Strain moduli and band gaps decrease whereas the exfoliation energies increase, as we consider chalcogenides going down the periodic column, i.e., S to Se to Te. Trends found in Table 1 are explained qualitatively in terms of the covalency of bonding. The covalent bond density is examined specifically in the case of S–N and SNH showing a weakening S–N covalent bond with the addition of H. The band gaps and excitonic binding energies for the insulating 1D materials considered here are found to be larger than their respective bulk counterparts. All chalcogenides have negative band deformation potentials. Finally, we consider heterojunction band offsets for several 1D systems.

We propose that 1D chalcogenide-based materials may be isolated from the corresponding bulk materials, which are readily available, following the procedure used to successfully isolate graphene and single chains of carbon from graphene. Nano-opto-electronics is one promising application area for these materials. Specifically, Se and Te may be good visible and IR absorbers, respectively. Also, the use of 1D heterojunctions may allow the realization of optically pumped atomic-scale lasers. Overall, the present calculations provide a view of the properties of several chalcogenide-based single molecular chain systems, which are promising for electronic, opto-electronic and other applications.

## Figures and Tables

**Table 1:** Results from our calculations for extended one-dimensional (1D) materials. The 1D lattice constant (*a_o_*), the strain modulus (*E_sm_*) and the exfoliation energy (*E_exf._*) are reported in the first three columns. Specifically, the exfoliation energy is calculated using: *E_exf._* = *E_Bulk_*/*N_Bulk_* − *E*_1D_/*N*_1D_ where *N_Bulk_* and *N*_1*D*_ are the number of atoms in the bulk and 1D calculations, respectively. All the G_0_W_0_ band gap results (*E_gap_*) are reported below. Indirect gaps are reported in parentheses in the two cases where the indirect gap is lower than the direct gap. The last column includes the deviation of the direct gap versus strain.

Material	*a_o_* (Å)	*E_sm_* (eV/Å)	*E_exf._* (eV/at.)	*E_gap_* (eV)	*D_gap_* (eV/%)
S	4.404	6.78	0.08	4.73 (4.60)	−0.13
Se	4.966	4.32	0.20	3.05 (3.00)	−0.06
Te	5.682	2.78	0.27	2.41	−0.05
SiS_2_	5.588	10.56	0.07	6.91 (6.34)	−0.02
SiSe_2_	5.939	8.19	0.09	5.25 (4.92)	−0.11
C_6_H_4_	4.364	31.07	0.12	2.60	+0.11
SNH	4.700	3.10	0.14	5.14	−0.07
SN	4.556	5.06	0.05	N/A	N/A
C_4_H_2_	2.468	50.9	0.18	N/A	N/A

**Table 2:** For four heterojunctions, we report the valence and conduction band offsets.

Material	VBO (eV)	CBO (eV)
Te–Se	0.44	0.20
SiSe_2_–SiS_2_	1.00	0.42
SN–SNH_2_	1.93	3.21
Se–P–SiSe_2_	0.13	1.72
